# Wild plant species with broader precipitation niches exhibit stronger host selection in rhizosphere microbiome assembly

**DOI:** 10.1093/ismeco/ycad015

**Published:** 2024-01-10

**Authors:** Haikun Ma, Jinming Liu, Lidong Mo, Luisa M Arias-Giraldo, Meichun Xiang, Xingzhong Liu

**Affiliations:** State Key Laboratory of Medicinal Chemical Biology, Key Laboratory of Molecular Microbiology and Technology of the Ministry of Education, Department of Microbiology, College of Life Sciences, Nankai University, Tianjin 300071, China; State Key Laboratory of Medicinal Chemical Biology, Key Laboratory of Molecular Microbiology and Technology of the Ministry of Education, Department of Microbiology, College of Life Sciences, Nankai University, Tianjin 300071, China; Department of Environmental Systems Science, Institute of Integrative Biology, ETH Zurich (Swiss Federal Institute of Technology), Universitätsstrasse 16, Zurich 8092, Switzerland; Department of Microbial Ecology, Netherlands Institute of Ecology, Wageningen, PB 6708, The Netherlands; State Key Laboratory of Mycology, Chinese Academy of Sciences, Institute of Microbiology, Beijing 100101, China; University of Chinese Academy of Sciences, Beijing 100049, China; State Key Laboratory of Medicinal Chemical Biology, Key Laboratory of Molecular Microbiology and Technology of the Ministry of Education, Department of Microbiology, College of Life Sciences, Nankai University, Tianjin 300071, China; State Key Laboratory of Mycology, Chinese Academy of Sciences, Institute of Microbiology, Beijing 100101, China

**Keywords:** precipitation niche, rhizosphere microbiome, host selection strength, wild plant species

## Abstract

Plants actively recruit microbes from the soil, forming species-specific root microbiomes. However, their relationship with plant adaptations to temperature and precipitation remains unclear. Here we examined the host-selected and conserved microbiomes of 13 native plant species in the Xilingol steppe, Inner Mongolia, a semi-arid region in China. By calculating the global precipitation and temperature niches of these plants, considering plant phylogenetic distances, and analyzing functional traits, we found that these factors significantly influenced the rhizosphere microbiome assembly. We further quantified the strength of host selection and observed that plants with wider precipitation niches exhibited greater host selection strength in their rhizosphere microbiome assembly and higher rhizosphere bacterial diversity. In general, the rhizosphere microbiome showed a stronger link to plant precipitation niches than temperature niches. *Haliangium* exhibited consistent responsiveness to host characteristics*.* Our findings offer novel insights into host selection effects and the ecological determinants of wild plant rhizosphere microbiome assembly, with implications for steering root microbiomes of wild plants and understanding plant-microbiome evolution.

## Introduction

Wild plant species live in habitats with few disturbances from anthropogenic activities. Therefore, wild plant species may maintain a greater diversity of functional traits and more complex co-evolutionary relationships with their rhizosphere microbiomes [1]. Along the same lines, wild plant species presumably face more intense natural selective pressures, driving them to maximize positive microbe-to-host effects by exerting a higher level of control over microbiome assembly and activity [2]. This may subsequently influence plant adaptations to environments under various temperature and precipitation ranges. However, how the intensity of the selective pressure imposed by plant on their rhizosphere microbiome is related to plant’s adaptations to precipitation and temperature on a broader (global) scale is still largely unknown.

**Figure 1:**
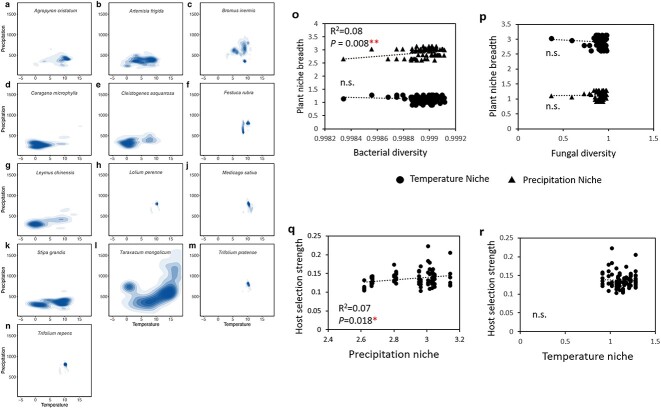
Global niche of 13 wild plant species (A–N), and their relationships with rhizosphere microbiome assembly (O–RO); for panel A–N, the *x*-axis is the temperature niche, *y*-axis is the precipitation niche; the niches are defined as the range between the highest value and the lowest value; for the statistical analysis, the variables of *x*-axis and *y*-axis were used separately; panels O and P show the linear regression between plant niche breadth with rhizosphere bacterial and fungal diversity, respectively; in each panel, dot represents plant temperature niche, triangle represents plant precipitation niche; panels Q and R show the linear regressions between plant host selection strength and plant precipitation niche, and plant temperature niche, respectively.

To begin to understand the relationships between plant niche breadth and plant rhizosphere microbiome assembly, we collected seeds of 13 common wild plant species native to the Inner Mongolia Xilingol steppe, one of the semi-arid areas in China, consistently restrained by water availability [3]. The soil is presumed to have a long history of coevolution with these wild plant species. We grew these wild plant species monoculturally under a controlled greenhouse environment to minimize potential biotic and abiotic interactions between plants and the environment, which may result in host selection pressure on their microbiomes [4] (see Materials and Methods in Supplementary Text). We assessed the global temperature and precipitation niches of these wild plant species based on their distributions (Fig. 1A–N, Supplementary Fig. S1, Supplementary Table S1) and investigated whether their microbiome assembly correlates with their global niches, plant phylogeny, and functional traits. Our results suggest that plants with a wider precipitation niche had stronger host selection and more diverse rhizobacterial communities.

## Results and discussion

Plant precipitation niche, but not the temperature niche, was significantly and positively related to plant rhizobacterial diversity (R^2^ = 0.08) and the host selection strength of plants over their rhizosphere microbiomes (R^2^ = 0.07, Fig. 1). Previous studies that examined the strengths of host selection effects have primarily relied on the explained variances obtained from the Permutational multivariate analysis of variance (PERMANOVA test [5]. However, this method provides a single value for all hosts, and the specific information on the host selection strengths of individual plant species is lacking. Although previous studies, such as Weinstein *et al*. [6] have discussed the significance of host selection effects, the explicit calculation of these strengths was not performed. However, Schneijderberg *et al*. [7] conducted a study where the strengths of host selection effects were calculated based on the number of altered amplicon sequencing variants (ASVs) by host plant species. In this study, we introduced a precise approach to represent host selection strengths by integrating the relative abundance and taxonomic diversity of host-selected ASVs, and comparing them to the relative abundance and number of conserved ASVs. Moreover, we observed that plants that had a broader precipitation niche also had higher rhizobacterial diversity. Higher taxonomical diversity in a community often indicates greater functional redundancy, facilitating plant colonization and adaptation to diverse environments [8]. A previous study [9] observed that drought had a stronger impact on soil bacterial communities, which subsequently influenced plant communities. Several other studies [10, 11] also reported that bacteria have a stronger influence on alleviating drought stress in plants compared to fungi. Therefore, the changes in bacterial communities under drought conditions may play a critical role in determining plant performance, whereas the role of fungal communities appears to be less influential.

**Figure 2:**
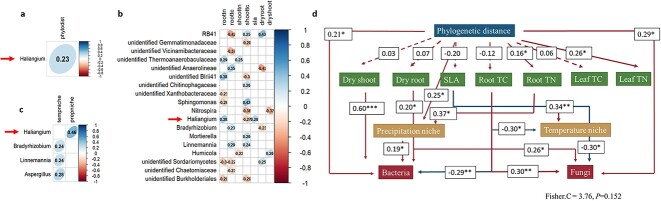
Genera that were highly correlated with plant phylogenetic distance (A), plant niches (B), plant functional traits (C), and structural equation models of plant phylogenetic distance, plant functional traits, plant niches, bacterial and fungal community composition (D); presented are genera with an average relative abundance higher than 0.01; in panels A, B, and C, the coefficients from Pearson correlations were shown in the cell, only significant correlations are shown; “Phylodist”: phylogenetic distances between wild plant species; “Tempniche”: plant actual temperature niche breadth; “Prepniche”: plant actual precipitation niche breadth; ``Dry shoot'': dry shoot mass; ``Dry root'': dry root mass; ``SLA'': specific leaf area; ``Root TC'': root total carbon; ``Root TN'': root total nitrogen; ``Leaf TC'': leaf total carbon; ``Leaf TN'': leaf total nitrogen. For panel D, positive paths are indicated by red lines, while negative paths are represented by blue lines; significant paths are shown as solid lines, while nonsignificant paths are depicted as dashed lines; the numbers on arrows indicate the effect size and significance of each path; only significant paths are displayed in the diagram, except for the relationships between plant phylogeny and plant functional traits; the paths between functional traits, plant niches, and bacterial and fungal communities are not shown.

Moreover, the results of structural equation modeling revealed that plant phylogeny had a direct influence on plant precipitation niche. It indirectly influenced plant precipitation niche through its influence on root nitrogen content, which in turn affected the rhizosphere bacterial community composition (Fig. 2, Supplementary Fig. S2). This observation indicates that although plant precipitation niche is strongly associated with the rhizosphere bacterial community, it is also determined by the interplay of plant functional traits and plant phylogeny. It is important to note that our study investigated plant species from the natural Inner Mongolia steppe. Therefore, for grassland plants in particular, adapting to precipitation may be of greater significance in their ability to adapt to the prevailing environmental conditions, including temperature. It is important to notice that there are only one precipitation/temperature niche, resulting in a total of only 13 variables. It is intriguing that, despite the limited number of variables, we consistently observed significant effects of plant precipitation niches on rhizobacterial diversity, overall community composition, and the strength of host selection.

The relative abundance of *Haliangium* was significantly positively correlated with plant phylogeny and plant precipitation niche and plant functional traits (Fig 2A–C). *Haliangium,* a genus of widely distributed bacteria, is known for its production of antifungal compounds: Haliangicin [12], which have been linked to plant growth-promoting effects in multiple studies [13–15]. In this study, *Haliangium* had an average relative abundance exceeding 0.01 in the rhizosphere of all wild plant species (Supplementary Fig. S3, S4). *Haliangium* were consistently selected by the host and positively associated with fast-growing life strategies, such as specific leaf area. These suggest that *Haliangium* may play a role in influencing host plants toward faster growth and facilitating their adaptation to arid environments. Our study contributes valuable insights for predicting the rhizosphere microbial composition of wild plant species, particularly in relation to the presence and influence of *Haliangium*.

 In this study, the plant species observed in the Inner Mongolia steppe are predominantly native species, rather than invasive ones. Together, these results suggest that plants with enhanced control over their microbiome could be utilized for ecosystem restoration and targeted manipulation of the microbiome in agricultural croplands [4]. It is very interesting to notice that wider distributer plants do not have higher precipitation/temperature niches; thus, we chose to use plant precipitation/temperature niches, as their global distribution may not accurately represent their niches (Fig. 1 and Supplementary Fig. S1). Future studies should consider investigating the rhizosphere microbiome of wild plant species across different natural biomes globally. Furthermore, validation experiments that involve manipulating rhizosphere microbial diversity, bacteria-to-fungi ratios, and cultivating plants that have distinct precipitation niches in different soil types could establish causal relationships between plant precipitation niches and the assembly of rhizosphere microbiomes.

## Supplementary Material

materials_and_methods19_12_23_ycad015

supplementary_materials25_01_24_ycad015

## Data Availability

All microbial data have been uploaded to the NCBI Sequence Read Archive under the BioProject accession number PRJNA884262. All data can be obtained in this manuscript or from the authors upon request.

## References

[1] Mariotte P , Mehrabi Z, Bezemer TM et al. Plant–soil feedback: bridging natural and agricultural sciences. Trends Ecol Evol 2018;33:129–42. 10.1016/j.tree.2017.11.005. 29241940

[2] Soldan R , Fusi M, Cardinale M et al. The effect of plant domestication on host control of the microbiota. Commun Biol 2021;4:1–9. 10.1038/s42003-021-02467-6. 34354230 PMC8342519

[3] Huang J , Bai Y, Jiang Y. Case study 3: Xilingol grassland, Inner Mongolia. In: Rangeland Degradation and Recovery in China's Pastoral Lands. Wallingford, UK: CABI, 2009, 120–35. 10.1079/9781845934965.0120.

[4] Cordovez V , Dini-Andreote F, Carrión VJ et al. Ecology and evolution of plant microbiomes. Annu Rev Microbiol 2019;73:69–88. 10.1146/annurev-micro-090817-062524. 31091418

[5] Xiong C , Zhu YG, Wang JT et al. Host selection shapes crop microbiome assembly and network complexity. New Phytol 2021;229:1091–104. 10.1111/nph.16890. 32852792

[6] Weinstein SB , Martínez-Mota R, Stapleton TE et al. Microbiome stability and structure is governed by host phylogeny over diet and geography in woodrats (*Neotoma* spp.). Proc Natl Acad Sci U S A 2021;118:e2108787118. 10.1073/pnas.2108787118. 34799446 PMC8617456

[7] Schneijderberg M , Cheng X, Franken C et al. Quantitative comparison between the rhizosphere effect of *Arabidopsis thaliana* and co-occurring plant species with a longer life history. ISME J 2020;14:2433–48. 10.1038/s41396-020-0695-2. 32641729 PMC7490400

[8] Louca S , Polz MF, Mazel F et al. Function and functional redundancy in microbial systems. Nat Ecol Evol 2018;2:936–43. 10.1038/s41559-018-0519-1. 29662222

[9] de Vries FT , Griffiths RI, Bailey M et al. Soil bacterial networks are less stable under drought than fungal networks. Nat Commun 2018;9:3033. 10.1038/s41467-018-05516-7. 30072764 PMC6072794

[10] Tufail MA , Ayyub M, Irfan M et al. Endophytic bacteria perform better than endophytic fungi in improving plant growth under drought stress: a meta-comparison spanning 12 years (2010–2021). Physiol Plant 2022;174:e13806. 10.1111/ppl.13806. 36271716

[11] Yang J , Kloepper JW, Ryu CM. Rhizosphere bacteria help plants tolerate abiotic stress. Trends Plant Sci 2009;14:1–4. 10.1016/j.tplants.2008.10.004. 19056309

[12] Sinong GF , Yasuda M, Nara Y et al. Distinct root microbial communities in nature farming rice harbor bacterial strains with plant growth-promoting traits. Front Sustain Food Syst 2021;4:629942. 10.3389/fsufs.2020.629942.

[13] Yan S , Zhao J, Ren T et al. Correlation between soil microbial communities and tobacco aroma in the presence of different fertilizers. Ind Crop Prod 2020;151:112454. 10.1016/j.indcrop.2020.112454.

[14] Daraz U , Li Y, Sun Q et al. Inoculation of *bacillus* spp. modulate the soil bacterial communities and available nutrients in the rhizosphere of vetiver plant irrigated with acid mine drainage. Chemosphere 2021;263:128345. 10.1016/j.chemosphere.2020.128345. 33297270

[15] Zhou X , Zhang J, Pan D et al. P-Coumaric can alter the composition of cucumber rhizosphere microbial communities and induce negative plant-microbial interactions. Biol Fertil Soils 2018;54:363–72. 10.1007/s00374-018-1265-x.

