# Supplementary material for: Wild plant species with broader precipitation niches exhibit stronger host selection in rhizosphere microbiome assembly
Source: ISME Commun. 2024 Jan 10;4(1):ycad015. doi: 10.1093/ismeco/ycad015 (PMC10910850; doi:10.1093/ismeco/ycad015)
Supplement: materials_and_methods19_12_23_ycad015 [file materials_and_methods19_12_23_ycad015.docx]

**Experimental set-up:**

Based on their common presence in the steppe community of the Inner Mongolia Steppe (annual precipitation: 140-400mm), the 13 wild plant species used in this study were *Agropyron cristatum* (ac, grass), *Bromus inermis* (bi, grass), *Lolium perenne* (lp, grass), *Cleistogenes squarrosa* (cs, grass), *Stipa grandis* (sg, grass), *Leymus chinensis* (lc, grass), *Festuca rubra* (fr, grass), *Taraxacum mongolicum* (tm, forb), *Artemisia frigida* (af, forb), *Caragana microphylla* (cm, legume), *Trifolium repens* (tr, legume), *Trifolium pratense* (tp, legume), and *Medicago sativa* (ms, legume). Instead of collecting live plant individuals, we gathered mature seeds of all these species directly from the wild areas of the Inner Mongolia Steppe in June 2021. Seeds from a single plant species were harvested from various individuals within the same geographical area, with precautions taken to ensure that individuals sharing a neighbourhood with the same plant species were not included in the sampling. The soil was collected at the depth from 5 to 20 cm in the natural grassland from Xilingol, Inner Mongolia. Soil samples were collected at random from five distinct points (> 200 m apart) within the steppe. After homogenizing all the soil samples, we sieved them using a 1 cm mesh size to remove coarse fragments and microarthropods. Seeds were surface sterilized in 3% sodium hypochlorite solution for 1 min, then rinsed and germinated on sterile glass beads in a growth chamber at 20 ℃ (16h/8h, light/dark). One-week-old seedlings were transplanted into individual pots (13 × 13 × 13 cm) in a monocultural setting, with five individuals per pot. Any seedlings that perished within the first week after transplantation were substituted with new individuals. Overall, a total of 78 pots were used for the experiment, consisting of six replicate pots for each of the 13 plant species. All pots were randomly distributed within the greenhouse and received regular watering throughout the experiment. Ten weeks after transplantation, all plants were harvested by clipping them at the soil level. The second fully expanded leaf from the top of each plant was then used to measure the leaf specific area. Plant roots were meticulously extracted from the soil, and 0.5 g of rhizosphere soil was obtained by gently sieving off the thin layer of soil adhered to the roots. The collected rhizosphere soil samples were then stored at -80 ℃ for subsequent amplicon sequencing.

**Plant actual niche width**

To obtain all the presence of 13 wild plant species, we downloaded the occurrence recordings from GBIF (Global Biodiversity Information Facility) (Detailed references are listed in Table S1, the geographical distribution of wild plants is depicted in Fig.S1). In addition, to ensure the relevance of the data, we only retained observations categorized as ‘HUMAN_OBSERVATION’ and recorded after 1950. Furthermore, in the subsequent statistical analysis, we focused on using temperature niche and precipitation niche instead of the geographical distribution range, as they are the most fundamental factors determining plant adaptations to the environment. To quantify the niche width of all these species, we extracted the annual mean temperature and mean annual precipitation data from the CHELSA database at the corresponding distribution points [1-3]. Therefore, for each species, we calculated the 95% quantile of the mean annual temperature and mean annual precipitation as the niche width for each plant species, referred to as the plant temperature niche and plant precipitation niche, respectively (**Fig.1**).

**Plant functional traits**

Roots were washed through a sieve with a 2 mm mesh, plant shoots and roots were dried in an oven at 60 ℃ for three days and subsequently weighed for dry shoot mass (Dry shoot) and dry root mass (Dry root). Specific leaf area (SLA) was determined by dividing the one-side leaf area by its dry mass. The dried leaves and roots were crushed, and then passed through a 0.15 mm mesh sieve for carbon and nitrogen measurements. Leaf total carbon (leafTC), leaf total nitrogen (leafTN), root total carbon (rootTC), and root total nitrogen (rootTN) were gauged using the Elemental Analyzer (Vario MACRO cube, Elementa).

**Microbial DNA extraction and sequencing**

DNA was extracted from 0.25 g rhizosphere soil using the PowerSoil DNA isolation kit (Qiagen, Hilden, Germany) following the manufacturer’s instructions. We amplified bacterial and fungal DNA in duplicate polymerase chain reaction (PCR) reactions using bar-coded primers. For bacteria, the primers 338F/806R (5'-ACTCCTACGGGAGGCAGCA-3'/5'-GGACTACHVGGGTWTCTAAT-3') targeting the V3 + V4 region of the 16S rRNA gene were used [4]. For fungi, the primers ITS2F/ITS2R (5'-GCATCGATGAAGAACGCAGC-3'/5'-TCCTCCGCTTATTGATATGC-3') targeting the intergenic transcribed spacer (ITS1) region were used [5]. High-throughput sequencing was conducted by Biomarker Tech (Beijing, China) using an Illumina Novaseq 6000 (Illumina, CA, USA).

**Bioinformatic analysis of plant rhizosphere microbiomes**

After sequencing, the demultiplexed paired-end fastq files were subjected to further analysis. Primer removal and quality trimming of the sequences were implemented by “cutadapt” [6]. Merging and quality filtering of the reads was performed using an error-corrected amplicon sequence variant (ASV) approach implemented in DADA2 v1.8 [7]. Taxonomic assignment was conducted using the naïve Bayesian classifier implemented in DADA2, with SILVA 138.1 and UNITE 2022.11.29 databases for bacteria and fungi, respectively. ASVs with less than 10 counts were excluded from the analysis and the taxonomical assignment was subjected to a quality check to make sure only pertinent categories were kept. Specifically, for bacteria, ASVs assigned to "eukaryota," "archaea," "chloroplast," and "mitochondria" were removed. For fungi, ASVs assigned as "Aphelidiomycota," "Zoopagomycota," and "Fungi_phy_Incertae_sedis" were excluded. The resulting ASV table was rarefied using Microbiome Analyst, and then was used for subsequent analysis. [8].

**Statistical analysis**

The sequencing data were normalized using the total sum scaling method (TSS) [9]. Representative sequences of ITS1 from each plant species were downloaded from NCBI [10]. Subsequently, the sequences were aligned separately using MUSCLE [11], and a Neighbour Joining tree was constructed in MEGA [12]. Mantel tests were conducted to examine the correlations between the plant phylogenetic tree and cluster trees of bacterial and fungal communities. Linear regressions were utilized to assess the relationships between [plant niche and rhizosphere microbial diversity], [host selection strength with plant global niches], [plant phylogenetic distances with Bray-Curtis dissimilarities of microbial communities]. Pearson correlations, visualized in the heatmap, were performed to explore the relationships between genera/ASVs with a relative abundance greater than 0.001 and plant niche, plant functional traits, and plant phylogeny (absolute pairwise differences of genera/ASVs between plant species were used).

**Host selection strength per plant species:**

$$host selection strength=\frac{\left( N_{changed}\times R_{changed} \right)\times D_{changed}}{(N_{conserved}\times R_{conserved})}$$

For the microbial communities of each plant species, ASVs were divided into conserved ones, and host selected ones. $N_{changed}$ is the number of ASVs that were influenced by specific plant species. $R_{changed}$ is the relative abundance of ASVs that were influenced by specific plant species. $D_{changed}$ is the Shannon diversity of the changed ASVs. $N_{conserved}$ is the number of conserved ASVs among all wild plant species present in the sample. $R_{conserved}$ is the relative abundance of the conserved ASVs in the sample. Conserved ASVs among all wild plant species were identified as those that did not show a significant influence by plant species identity based on one-way ANOVA. The range of this equation depends on the number of host-selected ASVs and the conserved ASVs across all host plant species. Since there are ASVs that remain unaffected by host identity, this equation is not associated with scenarios where the denominator equals zero.

The package 'piecewiseSEM' in R was utilized to perform Structural Equation Modeling (SEM) in order to examine the hypothetical causal relationships between plant phylogenetic distance, plant functional traits, plant niches, and root microbial communities [13]. In the SEM analysis, all variables were absolute pairwise variances between samples. Only the significant paths were depicted in the SEM model, and the relationships between plant functional traits were not shown to provide clarity to the model.

**References:**

1. Karger, DN, Wilson, AM, Mahony, C, Zimmermann, NE, Jetz, W. Global daily 1 km land surface precipitation based on cloud cover-informed downscaling. Sci Data. 2021; 8:307.
2. Karger, DN, Conrad, O, Böhner, J, Kawohl, T, Kreft, H, Soria-Auza, RW, *et al.* Climatologies at high resolution for the Earth land surface areas. Sci Data. 2017a; 4.
3. Karger DN, Conrad, O, Böhner, J, Kawohl, T, Kreft, H, Soria-Auza, RW, *et al.* Data from: Climatologies at high resolution for the earth’s land surface areas. Dryad Digital Repository. 2017b.
4. Caporaso, JG, Lauber, CL, Walters, WA, Berg-Lyons, D, Huntley, J, Fierer, N, *et al.* Ultra-high-throughput microbial community analysis on the Illumina HiSeq and MiSeq platforms. ISME J. 2012; 6:1621-1624.
5. Bengtsson‐Palme, J, Ryberg, M, Hartmann, M, Branco, S, Wang, Z, Godhe, A, *et al.* Improved software detection and extraction of ITS1 and ITS 2 from ribosomal ITS sequences of fungi and other eukaryotes for analysis of environmental sequencing data. Methods Ecol Evol*.* 2013; 4:914-919.
6. Martin, M. Cutadapt removes adapter sequences from high-throughput sequencing reads. EMBnet J. 2011; 17:10-12.
7. Callahan, BJ, McMurdie, PJ, Rosen, MJ, Han, AW, Johnson, AJ, Holmes, SP. DADA2: High-resolution sample inference from Illumina amplicon data. Nat Methods. 2016; 13:581-583.
8. Chong, J, Liu, P, Zhou, G, Xia, J. Using MicrobiomeAnalyst for comprehensive statistical, functional, and meta-analysis of microbiome data. Nat Protoc. 2020; 15:799-821.
9. Weiss, S, Xu, ZZ, Peddada, S, Amir, A, Bittinger, K, Gonzalez, A, Lozupone, C, et al. Normalization and microbial differential abundance strategies depend upon data characteristics. Microbiome. 2017; 5:1-18.
10. Sayers, EW, Bolton, EE, Brister, JR, Canese, K, Chan, J, Comeau, DC, *et al.* Database resources of the national center for biotechnology information. Nucleic Acids Res. 2022; 50:D20-D26.
11. Edgar, RC. MUSCLE: multiple sequence alignment with high accuracy and high throughput. Nucleic Acids Res. 2004; 32:1792-1797.
12. Hall, BG. Building phylogenetic trees from molecular data with MEGA. Mol Biol Evol. 2013; 30:1229-1235.
13. Lefcheck, JS. piecewiseSEM: Piecewise structural equation modelling in r for ecology, evolution, and systematics. Methods Ecol Evol. 2016; 7:573-579.
