# Supplementary material for: Wild plant species with broader precipitation niches exhibit stronger host selection in rhizosphere microbiome assembly
Source: ISME Commun. 2024 Jan 10;4(1):ycad015. doi: 10.1093/ismeco/ycad015 (PMC10910850; doi:10.1093/ismeco/ycad015)
Supplement: supplementary_materials25_01_24_ycad015 [file supplementary_materials25_01_24_ycad015.docx]

***Supplementary information***

**Wild plant species with broader precipitation niches exhibit stronger host selection in rhizosphere microbiome assembly**

**Table S1** References for calculating realised niches of wild plant species.

| Binomial | Reference |
| --- | --- |
| *Medicago sativa* L. | GBIF.org (31 March 2023) GBIF Occurrence Download https://doi.org/10.15468/dl.2mfv58 |
| *Trifolium pratense* L. | GBIF.org (31 March 2023) GBIF Occurrence Download https://doi.org/10.15468/dl.6kae7e |
| *Trifolium repens* L. | GBIF.org (31 March 2023) GBIF Occurrence Download https://doi.org/10.15468/dl.89kq9s |
| *Caragana microphylla* Lam. | GBIF.org (31 March 2023) GBIF Occurrence Download https://doi.org/10.15468/dl.jy3wxq |
| *Artemisia frigida* Willd. | GBIF.org (31 March 2023) GBIF Occurrence Download https://doi.org/10.15468/dl.27dr5u |
| *Taraxacum mongolicum* Hand.-Mazz. | GBIF.org (31 March 2023) GBIF Occurrence Download https://doi.org/10.15468/dl.m7d6nj |
| *Festuca rubra* L. | GBIF.org (31 March 2023) GBIF Occurrence Download https://doi.org/10.15468/dl.83yaja |
| *Leymus chinensis* (Trin.) Tzvelev | GBIF.org (31 March 2023) GBIF Occurrence Download https://doi.org/10.15468/dl.xdds8m |
| *Stipa grandis* P.A.Smirn. | GBIF.org (31 March 2023) GBIF Occurrence Download https://doi.org/10.15468/dl.nqnh7g |
| *Cleistogenes squarrosa* (Trin.) Keng | GBIF.org (31 March 2023) GBIF Occurrence Download https://doi.org/10.15468/dl.mwqzvx |
| *Lolium perenne* L. | GBIF.org (31 March 2023) GBIF Occurrence Download https://doi.org/10.15468/dl.9e9epb |
| *Bromus inermis* Leyss. | GBIF.org (31 March 2023) GBIF Occurrence Download https://doi.org/10.15468/dl.475b2g |
| *Agropyron cristatum* (L.) Gaertn. | GBIF.org (31 March 2023) GBIF Occurrence Download https://doi.org/10.15468/dl.hva977 |

**Fig.S1.** Maps showing the global distribution of 13 wild plant species.





**
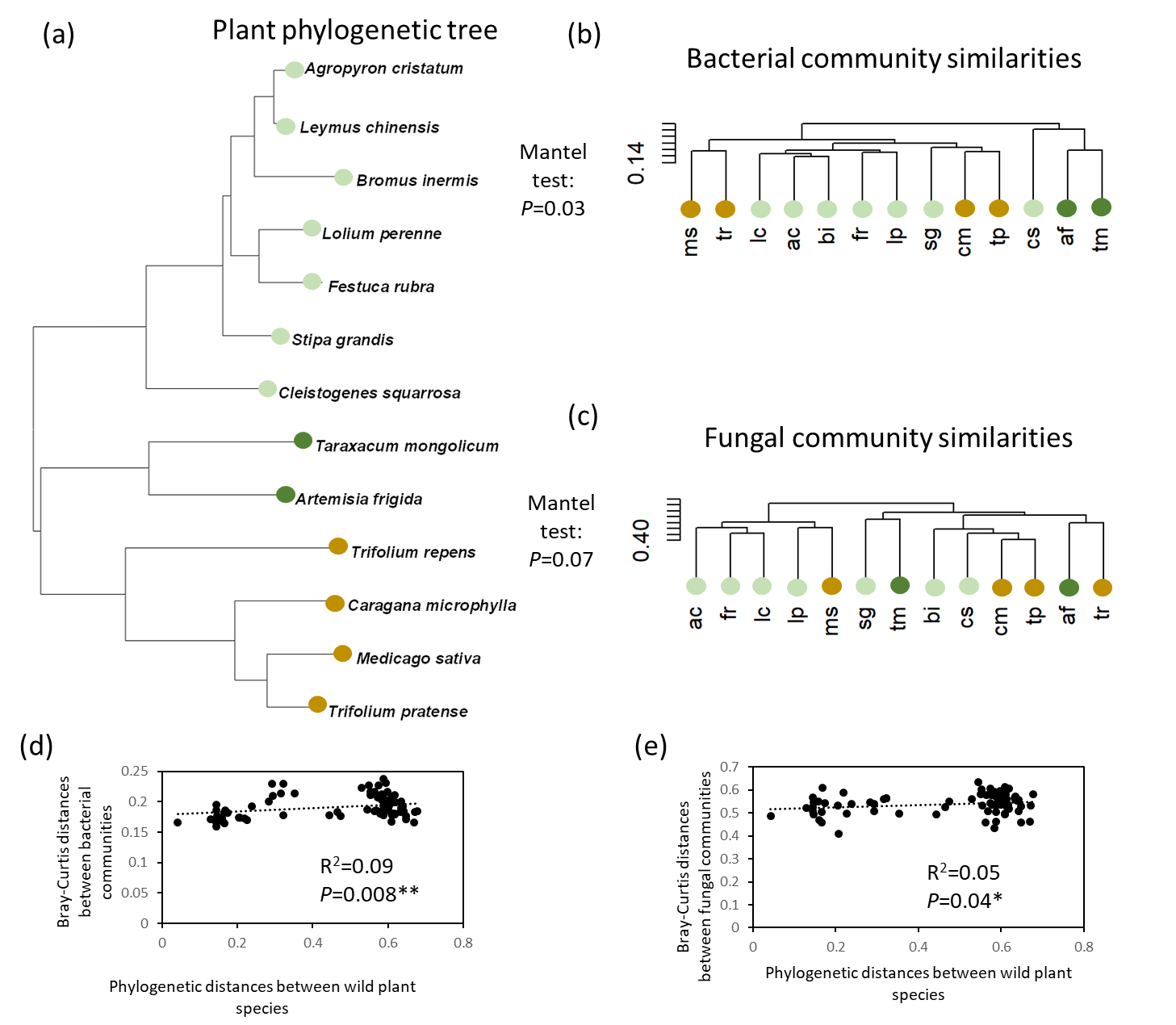
Fig.S2** The relationship between host plant phylogeny and the similarities of the rhizosphere microbiome community. (a) shows the phylogenetic tree of wild plant species. (b) and (c) show the dendrograms of the rhizosphere bacterial and fungi communities. In panels (a) to (c), the results of the mantel test between plant phylogenetic tree and cluster trees of bacterial and fungal communities were shown. The colour scheme represents different plant functional groups: light green represents grasses, dark green represents forbs, and brown represents legumes. (d) and (e) show the linear regressions between plant phylogenetic distances and Bray-Curtis dissimilarities of rhizosphere bacterial (P<0.05) and fungal (P<0.05) communities.


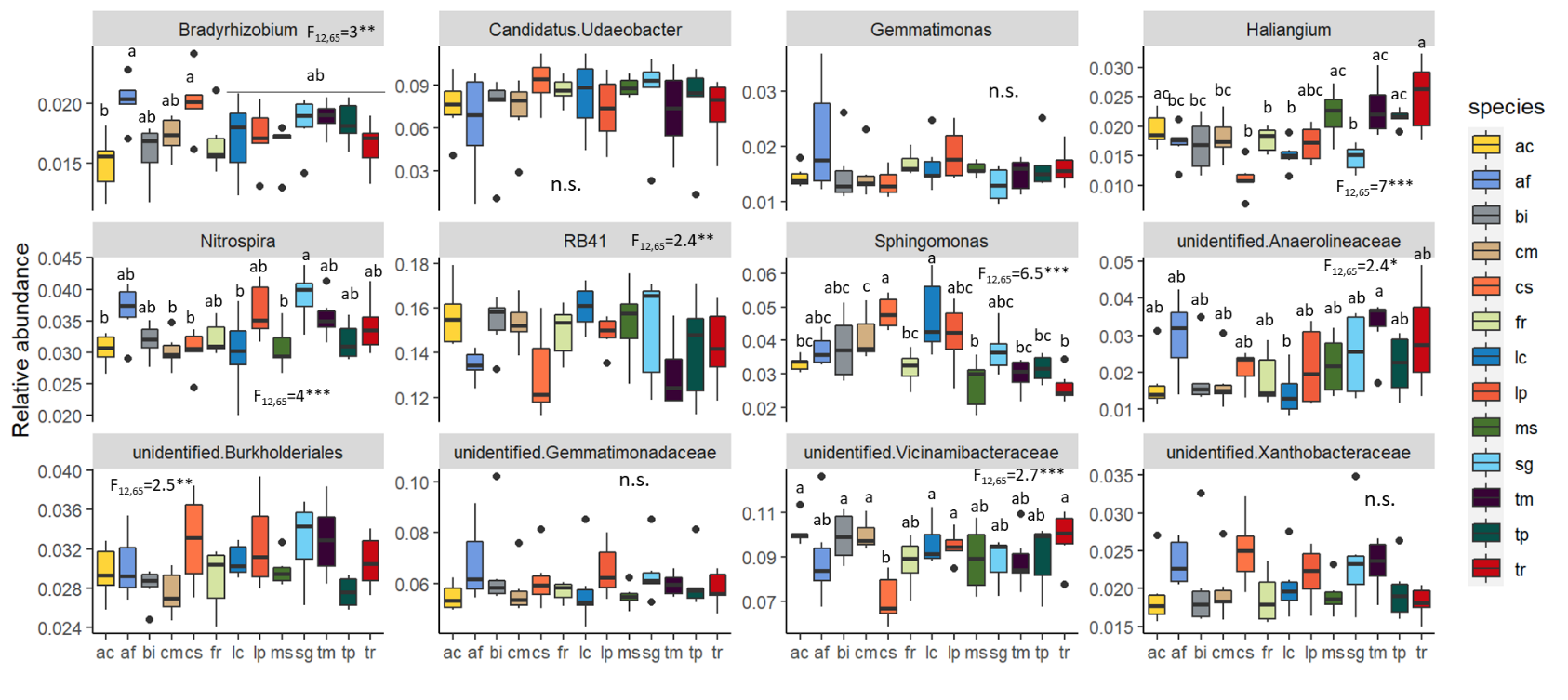
**Fig.S3**  The top 12 bacterial genera have the highest relative abundance among all wild plant species. For each bacterial genus, a one-way ANOVA was used to test the significant differences among wild plant species. For bacterial genera that significantly differ among wild plant species, the F value was shown in the panel, with *,**,*** indicating significance at 0.05, 0.01, 0.001, respectively. Different letters above bars indicate a significant difference between bars. The abbreviations of plant species are described in the materials and methods.


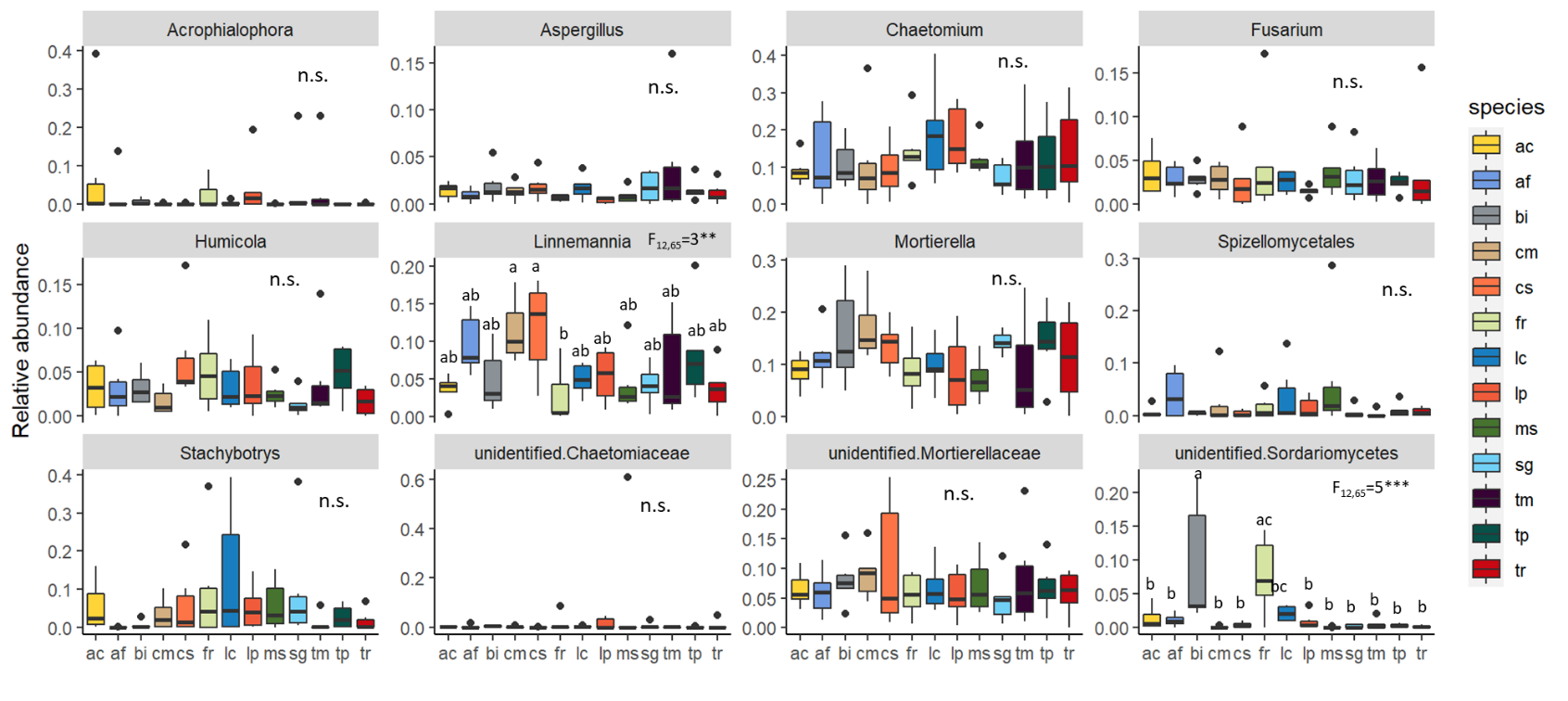
**Fig.S4** The top 12 fungal genera have the highest relative abundance among all wild plant species. For each fungal genus, a one-way ANOVA was used to test the significant differences among wild plant species. For fungal genera that significantly differ among wild plant species, the F value was shown in the panel, with *,**,*** indicating significance at 0.05, 0.01, 0.001, respectively. Different letters above bars indicate a significant difference between bars. The abbreviations of plant species are described in the materials and methods.
